# Clinicopathological characteristics and prognosis of young patients aged ≤45 years old with non-small cell lung cancer

**DOI:** 10.1515/med-2023-0684

**Published:** 2023-03-29

**Authors:** Jingjing Xia, Hong Li, Ruirui Zhang, Jipeng Wang

**Affiliations:** Department of Medical Examination, The Affiliated Huai’an No. 1 People’s Hospital of Nanjing Medical University, Huai’an, Jiangsu 223300, P.R. China; Department of Pathology, The Affiliated Huai’an No. 1 People’s Hospital of Nanjing Medical University, Huai’an, Jiangsu 223300, P.R. China; Department of Respiration, The Affiliated Huai’an No. 1 People’s Hospital of Nanjing Medical University, No. 1, Huanghexi Road, Huaiyin District, Huai’an, Jiangsu 223300, P.R. China

**Keywords:** young patients, non-small cell lung cancer, clinicopathological characteristics, survival risk factors, prognosis

## Abstract

Lung cancer is rare in young people, but the incidence and mortality are on the rise. We retrospectively analyzed the data of young patients aged ≤45 years diagnosed as lung cancer in our hospital from 2014 to 2021. The purpose was to explore the clinicopathological characteristics of young patients, and the risk factors affecting overall survival (OS) time. The results showed that the young patients were mainly female, had no smoking history, asymptomatic at initial diagnosis, with a high proportion of adenocarcinoma and stage I–II. We divided all patients into two groups according to age and found that the proportion of stage I–II in 18–35 years group was significantly higher than that in 36–45 years group (*P* = 0.021). The main manifestation of tumor was ground glass opacity (GGO) in 18–35 years group, while most showed non-GGO in 36–45 years group (*P* = 0.003). The proportion of minimally invasive adenocarcinoma was higher in 18–35 years group, while the invasive adenocarcinoma was higher in 36–45 years group (*P* = 0.004). Univariate analysis showed that asymptomatic, stage I–II, surgery, women, with few or no metastatic organs had longer OS. Multivariate analysis showed that the independent factors affecting the OS of young patients were tumor stage and more metastatic organs.

## 1 Introduction

Lung cancer is a very common malignant tumor in China, and it is also the primary cause of tumor-related death [1]. Non-small cell lung cancer (NSCLC) accounts for 85% of all lung cancer. Lung cancer mostly occurs in the elderly, and the median age of onset of lung cancer is 70 years old [2]. Young people with lung cancer are relatively rare, and NSCLC in young individuals with only <6% of all NSCLC patients being ≤45 years old [3]. There is a trend of increasing incidence of lung cancer among young adults in recent years [4,5], and the mortality rate also has an upward trend [6]. However, there are few studies on young patients with lung cancer. This study retrospectively analyzed 298 cases of NSCLC in young patients diagnosed in our hospital from 2014 to 2021, and explored the clinicopathological characteristics of young patients with lung cancer, the differences between different age subgroups, and risk factors affecting overall survival (OS).

## 2 Materials and methods

1. The clinical data of patients diagnosed with NSCLC aged 18–45 years in our hospital from January 2014 to December 2021 were retrospectively analyzed, with a total of 298 cases. All cases were confirmed by pathology. According to the latest classification of adenocarcinoma, carcinoma *in situ* was excluded [7]. Adenocarcinoma includes minimally invasive adenocarcinoma (MIA) and invasive adenocarcinoma (IA) in this study. Tumor staging was mainly based on the classification criteria of tumors, lymph nodes, and metastases (TNM) in the 8th editions of lung cancer. The following data were collected: gender, age, clinical feature, pathological type, TNM stage, lung CT manifestation, treatment, distant metastasis, etc. Around 276 patients were followed up until June 2022 or the date of death. This study was approved by the Ethic Committee of Huai’an First People’s Hospital (No. YW-2021-022-01).

2. All statistical analyses were performed using the SPSS19.0 software. Qualitative variables were described using counts and percent and compared by the Chi-square test. Kaplan–Meier method with log-rank tests were used for OS comparisons in univariate analysis. Multivariate analysis of survival was performed using the Cox proportional hazards regression to identify independent prognostic factors. The level of significance was set as 5%. The true effect of univariate analysis is easily covered by the influence of confounding factors, so we take the results of multivariate analysis as an independent risk factor affecting survival.

## 3 Results

### 3.1 Clinical characteristics of all young patients with NSCLC

A total of 298 young patients with lung cancer were included in the instant study, most of whom were female (65.1%) and had no smoking history (87.2%). The most common pathological type is adenocarcinoma (90.0%), including MIA (28.9%) and IA (61.1%). In addition, squamous cell carcinoma accounted for 4.7% and other types accounted for 5.3%. In terms of CT manifestations of tumors, 37.6% of cases showed as ground glass opacity (GGO), and non-GGO (solid nodule or mass or other) accounted for 62.4%. At the initial visit, most patients were asymptomatic (60.7%), 29.2% of them had cough/expectoration/sputum blood/asthma or fever, and other clinical symptoms were relatively rare. In respect of tumor staging, patients with stage I were the most (59.4%), followed by 71 patients with stage IV (23.8%), while patients with stage III and stage II accounted for 11.8 and 5.0%, respectively. Stage I–II was 192 (64.4%) in total, and stage III–IV was 106 (35.6%). Of all patients, 227 (76.2%) had no metastasis, 62 (20.8%) had 1–2 metastatic organs, and nine (3.0%) had three or more metastatic organs (Table 1). In 71 patients with stage IV, the most common distant metastasis site was bone (46.5%), followed by intrapulmonary (43.7%), pleural (29.6%), and brain (14.1%) (Table 2).

**Table 1:** Comparison of clinicopathological findings of the young patients with NSCLC between 18–35 years old group and 36–45 years old group

	Total (*n* = 298)	18–35 years (*n* = 80)	36–45 years (*n* = 218)	*P*
Gender				
Male	104 (34.9%)	27 (33.8%)	77 (35.3%)	
Female	194 (65.1%)	53 (66.2%)	141 (64.7%)	0.801
TNM stage				
I–II	192 (64.4%)	60 (75.0%)	132 (60.6%)	
III–IV	106 (35.6%)	20 (25.0%)	86 (39.4%)	0.021*
Surgery				
Yes	217 (72.8%)	60 (75.0%)	157 (72.0%)	
No	81 (27.2%)	20 (25.0%)	61 (28.0%)	0.608
Smoking history				
Yes	38 (12.8%)	12 (15.0 %)	26 (11.9%)	
No	260 (87.2%)	68 (85.0%)	192 (88.1%)	0.481
CT manifestations				
GGO	112 (37.6%)	41 (51.3%)	71 (32.6%)	
Non-GGO	186 (62.4%)	39 (48.7%)	147 (67.4%)	0.003*
Symptoms at diagnosis				
Asymptomatic	181 (60.7%)	55 (68.8%)	126 (57.8 %)	
Symptomatic	117 (39.3%)	25 (31.2%)	92 (42.2%)	0.086
Histologic type				
MIA	86 (28.9%)	34 (42.5%)	52 (23.9%)	
IA	182 (61.1%)	36 (45.0%)	146 (66.9%)	
SqCC	14 (4.7%)	4 (5.0%)	10 (4.6%)	
Other types	16 (5.3%)	6 (7.5%)	10 (4.6%)	0.004*
Number of distant metastatic organs				
0	227 (76.2%)	63 (78.8%)	164 (75.2%)	
1–2	62 (20.8%)	12 (15.0%)	50 (23.0%)	
≥3	9 (3.0%)	5 (6.2%)	4 (1.8%)	0.059

MIA, minimally invasive adenocarcinoma; IA, invasive adenocarcinoma; SqCC, squamous cell carcinoma; other types, undifferentiated or other mixed NSCLC. *Indicates significant difference. *P*-value was the Chi-square test result of each factor between the two groups.

**Table 2:** Metastases of young patients at stage IV (*n* = 71)

Distant metastatic site	Number	Proportion (%)
Bone	33	46.5
Intrapulmonary	31	43.7
Pleura	21	29.6
Brain	10	14.1
Adrenal gland	7	9.9
Liver	6	8.5
Pericardium	5	7.0
Kidney	3	4.2
Pancreas	1	1.4
Muscle	1	1.4
Skin	1	1.4
Uterine appendage	1	1.4

### 3.2 Comparison of clinicopathological findings between 18–35 years old group and 36–45 years old group

We divided all patients into two groups according to age, 18–35 years old group and 36–45 years old group. The clinicopathological characteristics of the two groups were compared. The results revealed that there were statistical differences in TNM stage, pathological types, and lung CT manifestations of tumors between the two groups. The proportion of stage I–II in the 18–35 years old group was significantly higher than that in the 36–45 years old group (75.0% vs 60.6%, *P* = 0.003). There were statistical differences in the distribution of pathological types between the two groups (*P* = 0.004). Adenocarcinoma accounted for the most in both groups. However, the proportion of MIA in the 18–35 years old group was higher than that in the 36–45 years old group (42.5% vs 23.9%), while the proportion of IA in the 36–45 years old group was higher (66.9% vs 45.0%). Lung CT manifestation in the two groups had statistical difference (*P* = 0.003). The 18–35 years old group mainly showed GGO, while those in the 36–45 years old group mainly showed non-GGO. There was no significant difference between the two groups in gender, smoking history, surgery or not, the number of distant metastatic organs, and clinical manifestations at the initial diagnosis (Table 1).

### 3.3 Comparison of clinicopathological findings between 2014–2017 and 2018–2021

In order to study whether the characteristics of lung cancer in young patients have changed in recent years, we divided all patients into two groups for analysis according to the year of diagnosis: 2014–2017 and 2018–2021. We found that the proportion of patients in stage I–II (71.9% vs 41.9%), asymptomatic (70.5% vs 31.1%), MIA (37.9% vs 1.4%), GGO (48.2% vs 5.4%), and surgical treatment (78.1% vs 56.8%) during 2018–2021 was significantly higher than that in 2014–2017 (*P* < 0.05). The number of distant metastatic organs was significantly lower than that in the 2014–2017 group (*P* < 0.05) (Table 3).

**Table 3:** Comparison of clinicopathological findings of the young patients with NSCLC between 2014–2017 and 2018–2021

	2014–2017 (*n* = 74)	2018–2021 (*n* = 224)	*P*
Gender			
Male	29 (39.2%)	75 (33.5%)	
Female	45 (60.8%)	149 (66.5%)	0.372
TNM stage			
I–II	31 (41.9%)	161 (71.9%)	
III–IV	43 (58.1%)	63 (28.1%)	<0.001*
Smoking history			
Yes	12 (16.2%)	26 (11.6%)	
No	62 (83.8%)	198 (88.4%)	0.303
Surgery			
Yes	42 (56.8%)	175 (78.1%)	
No	32 (43.2%)	49 (21.9%)	<0.001*
Number of distant metastatic organs			
0	46 (62.2%)	181 (80.8%)	
1–2	24 (32.4%)	18 (8.0%)	
≥3	4 (5.4%)	5 (2.2%)	<0.001*
Histologic type			
MIA	1 (1.4%)	85 (37.9%)	
IA	59 (79.7%)	123 (54.9%)	
SqCC	8 (10.8%)	6 (2.7%)	
Other types	6 (8.1%)	10 (4.5%)	<0.001*
CT manifestations			
GGO	4 (5.4%)	108 (48.2%)	
Non-GGO	70 (94.6%)	116 (51.8%)	<0.001*
Symptoms at diagnosis			
Asymptomatic	23 (31.1%)	158 (70.5%)	
Symptomatic	51 (68.9%)	66 (29.5%)	<0.001*

MIA, minimally invasive adenocarcinoma; IA, invasive adenocarcinoma; SqCC, squamous cell carcinoma; other types, undifferentiated or other mixed NSCLC. *Indicates significant difference. *P*-value was the Chi-square test result of each factor between the two groups.

### 3.4 Univariate and multivariate analysis of risk factors affecting OS

**Figure 1:**
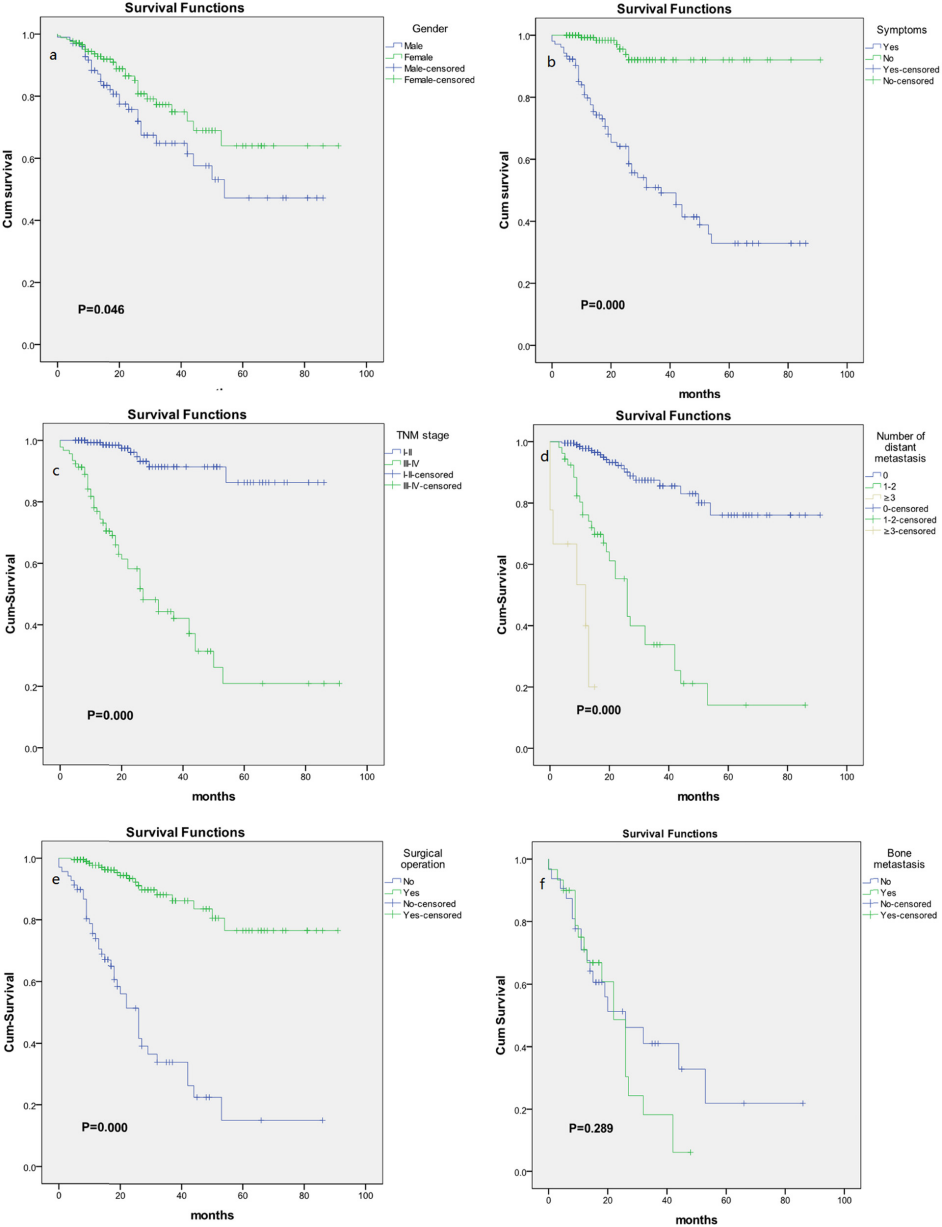
Survival curves in young patients of different groups: (a) different gender groups, (b) groups of symptomatic and asymptomatic, (c) groups of stage I–II and stage III–IV, (d) groups of different number of metastasis organs, (e) groups of surgery or not, and (f) groups of bone metastasis or not in stage IV patients.

Among the 298 cases of NSCLC in this study, 276 cases were followed up. We used Kaplan–Meier method and log rank tests to draw the survival curve and conduct univariate analysis. Then Cox multivariate analysis was performed for statistically significant factors. We found that females had better prognosis than males (*P* = 0.046).Asymptomatic patients had longer OS than symptomatic patients (*P* < 0.001). Patients in stage I–II had better prognosis than stage III–IV (*P* < 0.001). Patients receiving surgery have had better prognosis than those without surgery (*P* < 0.001). Patients without distant metastasis or with 1–2 metastatic organs had longer OS than patients with metastatic organs ≥3 (*P* < 0.001). The survival curves are shown in Figure 1. There were no differences in smoking, age group (18–35 and 36–45 years), and pathology type (adenocarcinoma and non-adenocarcinoma). Cox multivariate analysis showed that the independent risk factors affecting OS of young patients were TNM stage (HR = 3.314, 95% CI 1.132–9.702, *P* = 0.029) and the number of distant metastatic organs (HR = 2.413, 95% CI 1.116–5.217, *P* = 0.025) (Table 4).

**Table 4:** Univariate and multivariate analysis for risk factors of OS in young patients with NSCLC

	Univariate analysis		Multivariate analysis		
	*χ* ^2^	^#^ *P*	HR	95% CI	^##^ *P*
TNM stage	73.805	<0.001*	3.314	1.132–9.702	0.029*
I–II					
III–IV					
Number of distant metastatic organs	121.820	<0.001*	2.413	1.116–5.217	0.025*
0					
1–2					
≥3					
Gender	3.997	0.046*	0.743	0.424–1.300	0.298
Male					
Female					
Symptoms at diagnosis	51.353	<0.001*	0.392	0.133–1.155	0.089
Symptomatic					
Asymptomatic					
Surgery	79.932	<0.001*	1.137	0.410–3.155	0.805
Yes					
No					
Age	2.155	0.142	—	—	—
18–35 years					
36–45 years					
Adenocarcinoma or not	1.25	0.264	—	—	—
Yes					
No					
Smoking history	0.393	0.531	—	—	—
Yes					
No					

^#^ *P*-value was the result of univariate analysis. ^##^ *P* was the result of multivariate analysis. *Indicates significant difference. HR, hazard ratio; CI, confidence interval.

### 3.5 Survival analysis of stage IV patients with bone metastasis or not

There were 71 patients with stage IV in this study. The most common distant metastasis site is bone (46.5%), followed by intrapulmonary (43.7%), pleural (29.6%), and brain (14.1%) (Table 2). Patients with stage IV were divided into two groups according to whether there was bone metastasis, the results showed that there was no statistical difference in OS between the two groups, *P* = 0.289 (Figure 1).

## 4 Discussion

Recent studies show that occupations with high exposure risk, smoking, and family history of lung cancer are risk factors for lung cancer in young people [8,9]. Young patients with lung cancer have unique clinicopathological and gene mutation characteristics, such as common in women, no smoking history, mainly adenocarcinoma, with advanced TNM stage, and prevalence of targeted genetic alterations such as ALK gene and EGFR gene mutations. The prognosis of young patient with lung cancer is often poor, but these views are controversial [6,8,10–17]. This situation may be related to different research objects and age cut-off, or it may be related to different races of the population. For example, some studies choose all young patients with lung cancer, while others chose only NSCLC or adenocarcinoma in young adults. The age cut-off selected ranged from 40 to 50 years old. These differences will lead to different results. Young people under the age of 45 years old have distinctive genetic profile [18], which can be used as the age cut-off for young people with lung adenocarcinoma. Therefore, we used 45 years old as the age cut-off for young people with lung cancer in this study.

This study showed that females are in the majority, mainly adenocarcinoma (90.0%), and most patients have no smoking history, which is consistent with many literature reports [6,11,12]. The smoking rate of young people with lung cancer in this study is very low (12.8%), far lower than the literature report [19]. This may be related to the national tobacco control and the improvement of young people’s awareness about the harmful effects of tobacco, and the smoking rate of Chinese women is not high. But this proportion is basically consistent with another study, i.e., the smoking rate in lung cancer patients aged below 40 years old was 14.4% [2]. Although many young women have no smoking history, the high incidence of lung cancer may be related to inhalation of kitchen oil smoke or second-hand smoke [20]. Of course, the reasons in genomics await further in-depth research.

In terms of tumor stage, this study shows that young patients with lung cancer in stage I–II is more common (64.4%), and stage III–IV only accounts for 35.6%, which is different from other literatures that young patients are mainly in advanced stage [8]. We consider that this is related to the detection of many MIA in young adults in recent years. It can be seen from the grouping of diagnosis years that many MIA in the form of GGO were found from 2008 to 2021 (Table 3). Especially since the COVID-19 epidemic at the end of 2019, many asymptomatic young people underwent chest CT examination and screened a large number of GGO in the lung. Most of these lung cancers with GGO are MIA or early stage IA, which has led to an increase in the proportion of stage I–II patients in recent years.

There were statistical differences between the two groups (18–35 years old group and 36–45 years old group) in TNM stage, pathological type, and CT manifestation of the tumor. Compared with the 36–45 years old group, there are more patients with stage I–II and the tumors mainly showed as GGO in the 18–35 years old group. Although adenocarcinoma accounted for the most in both the groups, the proportion of MIA in the 18–35 years old group was relatively higher than in 36–45 years old group (42.5% vs 23.9%), while the proportion of IA in the 36–45 years old group was relatively higher (66.9% vs 45.0%). There was no significant difference between the two groups in gender, smoking history, surgery or not, the number of distant metastatic organs, and clinical manifestations. Therefore, we believe that patients in the 18–35 years old group had unique clinicopathological characteristics, including that lung lesions were more manifested as GGO, with early stage at the initial diagnosis, and the pathological type was mainly MIA.

Among 71 patients with stage IV, the most common metastasis site was bone (46.5%), followed by intrapulmonary (43.7%), pleural (29.6%), and brain (14.1%).The results were similar to one study on young patients with NSCLC [16], which shows the sites of metastases: bone (43%), intrapulmonary lesions (37%), pleura (30%), and brain (21%) metastases were the four most common metastases. Studies have shown that the survival time of lung cancer with bone metastasis is shorter than that of other metastasis sites [21,22]. We divided patients with stage IV into two groups according to whether there was bone metastasis and compared the OS time, but the results showed that there was no statistical difference between the two groups. The effect of bone metastasis on the prognosis of young patients with lung cancer in this study is inconsistent with the above literature, which may have three reasons. First, different sites and numbers of bone metastases have different effects on prognosis [23]. Second, the subjects of previous literature were all lung cancer patients without age limit, while this study was mainly aimed at young patients with lung cancer. Third, the small sample size of a single center may also have a certain impact on the results.

There were few relevant studies on the prognosis of young patients with lung cancer, and the obtained results have been controversial. A study [6] shows that a family history of cancer, symptoms at diagnosis, pathology, stage at diagnosis, and surgery were confirmed as independent prognostic factors in younger lung cancer patients. In another article published in 2017, 420 young patients with lung cancer from two Chinese hospitals from 2000 to 2003 were analyzed. Multivariate analysis did not show that pathological type was an independent risk factor affecting the survival of young patients with lung cancer [8]. In yet another US study, it was shown that male sex and non-adenocarcinoma histology were independent negative prognostic factors among the young. Moreover, compared with the total population, African Americans was a poor prognostic factor for young people [24]. Due to the limited number of samples in this group and the small number of patients with squamous cell carcinoma, we divided all young NSCLC into adenocarcinoma and non-adenocarcinoma groups, and there was no significant difference in survival time between the two groups. The inconsistency of these results may be related to the race of the subjects and the limited small sample size. Therefore, the influence of pathological types on the prognosis of young patients with lung cancer needs more research to verify.

The univariate analysis of this study shows that females, asymptomatic patients, and those who underwent surgery had longer survival time in young patients with lung cancer (*P* < 0.05), which is consistent with literature report [6]. In addition, the univariate analysis of this study shows that TNM stage and the number of distant metastatic organs are also important factors affecting OS of young patients with lung cancer. We included gender, surgery or not, TNM stage, the number of distant metastatic organs, and symptoms into Cox multifactor analysis, and finally concluded that the independent risk factors affecting OS of young patients with NSCLC were TNM stage (HR = 3.314, 95% CI 1.132–9.702, *P* = 0.029) and the number of distant metastatic organs (HR = 2.413, 95% CI 1.116–5.217, *P* = 0.025).

Smoking is a well-known risk factor that causes lung cancer and affects the prognosis of patients, but the results of this study show that the prognosis of young patients with lung cancer is not related to smoking history. This is confusing. However, in recent years, many reports show that young patients with lung cancer is mostly female and has no smoking history, so the natural development process of lung cancer in young patients cannot be completely explained by smoking. There may be other unknown reasons, such as genomic mutations, which need further research. This may also be related to the fact that many patients smoke passively or inhale more lampblack without enough attention, and the small sample size of a single center.

There are few literature on the impact of age on OS in young patients with lung cancer. All patients were divided into 18–35 years old group and 36–45 years old group due to the limitation of sample size in this study, and univariate analysis showed that there was no statistical difference in OS between the two groups. Therefore, whether the OS of young patients under 45 years old with NSCLC is affected by age needs further study.

This study also has some shortcomings: small sample size of a single center, lack of some other important factors collected, such as gene mutation, family history, etc. Young people are the backbone of the country and shoulder multiple responsibilities for family and society. Enough attention should be paid to young people in the face of the increasing incidence and mortality in young patients with lung cancer. However, there are few studies on young patients with lung cancer at present, especially on the risk factors affecting the survival time. This study can provide reference for more studies in the future, and also give some enlightenment to clinical work. Most young patients have no clinical symptoms at the first diagnosis. Recognizing the clinicopathological characteristics of young patients with lung cancer can reduce misdiagnosis and avoid late diagnosis. In addition, a large number of GGO have been detected in asymptomatic people in recent years, and most of them are MIA or early stage IA [25,26]. Adenocarcinoma with GGO has a good prognosis [27,28]. In our study, the CT findings of young patients with NSCLC in 2018–2021 group were mainly GGO, while the lung lesions of young patients diagnosed in 2014–2017 were mostly non-GGO. The patients in the 18–35 years old group were more likely to show GGO than those in the 36–45 years old group. Therefore, we believe that young patients with NSCLC tend to have GGO as the main CT manifestations in recent years. If this part of patients with early stage can be screened out and treated surgically, then these patients will have a very good prognosis and survival. At present, the recommended screening age for lung cancer in China is 50 years old. It is worth considering whether low-dose chest CT screening can be carried out in young people aged below 45 years, like once every 2–3 years. The purpose is to detect and treat young patients with lung cancer in the early stage, prolong survival time, and reduce the pain of individuals and families and the burden of the country caused by advanced lung cancer.

## 5 Conclusion

From 2018 to 2021, there were many asymptomatic young patients with NSCLC with stage I–II in the form of GGO. Among all young patients, most are females, with no smoking history, have no symptoms at the first diagnosis, mainly adenocarcinoma, and with stage I–II. Compared with the 36–45 years old group, patients in the 18–35 years old group have unique clinicopathological characteristics. The proportion of stage I–II in the 18–35 years old group is significantly higher than that in the 36–45 years old group. Lung cancer mainly manifested as GGO in the 18–35 years old group, while most of the lesions were non-GGO (solid nodules, masses, or other) in 36–45 years old group. Adenocarcinoma accounted for the most in both the groups, but the proportion of MIA in the 18–35 years old group was relatively higher than in 36–45 years old group, while the proportion of IA in the 36–45 years old group was relatively higher. The independent risk factors affecting the OS of young patients with NSCLC are TNM stage and the number of distant metastatic organs. There was no statistical difference in terms of OS whether there was bone metastasis in stage IV patients.
